# *N*-(2,4,6-Trimethyl­phen­yl)maleamic acid

**DOI:** 10.1107/S1600536809044754

**Published:** 2009-10-31

**Authors:** B. Thimme Gowda, Miroslav Tokarčík, Jozef Kožíšek, K. Shakuntala, Hartmut Fuess

**Affiliations:** a Department of Chemistry, Mangalore University, Mangalagangotri 574 199, Mangalore, India; b Faculty of Chemical and Food Technology, Slovak Technical University, Radlinského 9, SK-812 37 Bratislava, Slovak Republic; c Institute of Materials Science, Darmstadt University of Technology, Petersenstrasse 23, D-64287 Darmstadt, Germany

## Abstract

The mol­ecular structure of the title compound, C_13_H_15_NO_3_, is stabilized by a short intra­molecular O—H⋯O hydrogen bond within the maleamic unit. In the crystal, inter­molecular N—H⋯O hydrogen bonds link mol­ecules into zigzag chains propagating in [010].

## Related literature

For our sudies on the effect of ring- and side-chain substitutions on the crystal structures of amides, see: Gowda, Foro *et al.* (2009 ▶); Gowda, Tokarčík *et al.* (2009**a* ▶,b* ▶); Lo & Ng (2009 ▶). For hydrogen bonds in carboxylic acids, see: Leiserowitz (1976 ▶).
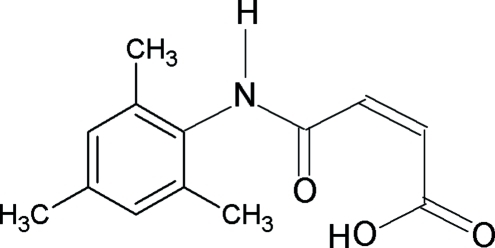


## Experimental

### 

#### Crystal data

C_13_H_15_NO_3_
*M* *_r_* = 233.26
Monoclinic, 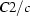

*a* = 10.8789 (3) Å
*b* = 11.9095 (2) Å
*c* = 20.1004 (5) Å
β = 103.014 (2)°
*V* = 2537.36 (10) Å^3^
*Z* = 8
Mo *K*α radiation
μ = 0.09 mm^−1^
*T* = 295 K
0.56 × 0.48 × 0.35 mm

#### Data collection

Oxford Diffraction Xcalibur Ruby Gemini diffractometer
Absorption correction: analytical (*CrysAlis Pro*; Oxford Diffraction, 2009 ▶) *T*_min_ = 0.933, *T*_max_ = 0.965
26868 measured reflections
2386 independent reflections
2029 reflections with *I* > 2σ(*I*)
*R*_int_ = 0.023

#### Refinement

*R*[*F* ^2^ > 2σ(*F* ^2^)] = 0.037
*wR*(*F* ^2^) = 0.102
*S* = 1.04
2386 reflections
159 parameters
H-atom parameters constrained
Δρ_max_ = 0.17 e Å^−3^
Δρ_min_ = −0.12 e Å^−3^

### 

Data collection: *CrysAlis Pro* (Oxford Diffraction, 2009 ▶); cell refinement: *CrysAlis Pro*; data reduction: *CrysAlis Pro*; program(s) used to solve structure: *SHELXS97* (Sheldrick, 2008 ▶); program(s) used to refine structure: *SHELXL97* (Sheldrick, 2008 ▶); molecular graphics: *ORTEP-3* (Farrugia, 1997 ▶) and *DIAMOND* (Brandenburg, 2002 ▶); software used to prepare material for publication: *SHELXL97*, *PLATON* (Spek, 2009 ▶) and *WinGX* (Farrugia, 1999 ▶).

## Supplementary Material

Crystal structure: contains datablocks I, global. DOI: 10.1107/S1600536809044754/bt5119sup1.cif

Structure factors: contains datablocks I. DOI: 10.1107/S1600536809044754/bt5119Isup2.hkl

Additional supplementary materials:  crystallographic information; 3D view; checkCIF report

## Figures and Tables

**Table 1:** Hydrogen-bond geometry (Å, °)

*D*—H⋯*A*	*D*—H	H⋯*A*	*D*⋯*A*	*D*—H⋯*A*
O2—H2*A*⋯O1	0.90	1.61	2.5037 (13)	174
N1—H1*N*⋯O3^i^	0.86	2.12	2.9587 (15)	165

Symmetry code: (i) 
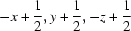
.

**Fig. 1.**
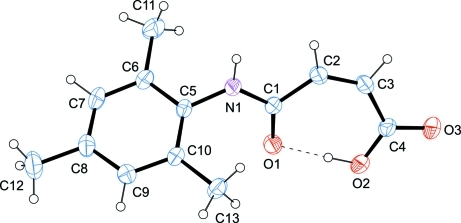
Molecular structure of (I) showing the atom labelling scheme. Displacement ellipsoids are drawn at the 30% probability level. H atoms are represented as small spheres of arbitrary radii.

**Fig. 2.**
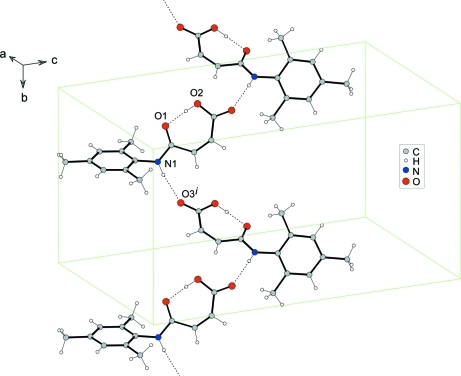
Part of crystal structure of (I) with two-dimensional framework generated by N—H···O hydrogen bonds, shown as dashed lines. Symmetry code (i): -x + 1/2, y + 1/2, -z + 1/2.

## supplementary crystallographic information

### Comment

As a part of studying the effect of ring and side chain substitutions on the crystal structures of amide derivatives (Gowda, Foro *et al.*, 2009; Gowda, Tokarčík *et al.*, 2009*a,b*), the crystal structure of *N*-(2,4,6-trimethylphenyl)-maleamic acid (I) has been determined. The conformations of the N–H and C=O bonds in the amide segment of the structure are *anti* to each other. Further, the conformation of the amide O atom is *anti* to the H atom attached to the adjacent C atom, while the carboxyl O atom is *syn* to the H atom attached to its adjacent C atom (Fig.1). The rare anti conformation of the C=O and O–H bonds of the acid group has been observed, similar to that obsrved in *N*-(2,6-dimethylphenyl)-maleamic acid (Gowda, Tokarčík *et al.*, 2009*a*) and *N*-phenylmaleamic acid (Lo & Ng, 2009), but contrary to the more general *syn* conformation observed for C=O and O–H bonds of the acid group in *N*-(2,4,6-trimethylphenyl)succinamic acid (Gowda, Foro *et al.*, 2009). The various modes of interlinking carboxylic acids by hydrogen bonds is described elsewhere (Leiserowitz, 1976).

The maleamic moiety includes a short intramolecular hydrogen O–H···O bond (Table 1). The C2–C3 bond length of 1.325 (2) Å clearly indicates the double bond character. The dihedral angle between the phenyl ring and the amido group –NHCO– is 58.3 (2)°. The mean plane through all the atoms of the maleamic moiety (N1, C1, C2, C3, C4, O1, O2 and O3) has a r.m.s. value of 0.06 Å, with the most deviating atom N1. In the crystal structure, the intermolecular N–H···O hydrogen bonds link the molecules into zigzag ribbons propagated in the [0 1 0] direction (Fig. 2).

### Experimental

The solution of maleic anhydride (0.025 mol) in toluene (25 ml) was treated dropwise with the solution of 2,4,6-trimethylaniline (0.025 mol) also in toluene (20 ml) with constant stirring. The resulting mixture was stirred for about 30 min and set aside for   additional 30 min at room temperature for the completion of reaction. The mixture was then treated with dilute hydrochloric acid to remove the unreacted 2,4,6-trimethylaniline. The resultant solid *N*-(2,4,6-trimethylphenyl)maleamic acid was filtered under suction and washed thoroughly with water to remove the unreacted maleic anhydride and maleic acid. It was recrystallized to constant melting point from ethanol. The purity of the compound was checked by elemental analysis and characterized by its infrared spectra. The single crystals used in X-ray diffraction studies were grown in an ethanol solution by slow evaporation at room temperature.

### Refinement

All H atoms were placed in calculated positions (C–H = 0.93 or 0.96 Å, N–H = 0.86 Å, O–H = 0.90 Å) and refined using a riding model. The *U*_iso_(H) values were set at 1.2*U*_eq_(C_aromatic_, N, O) or 1.5*U*_eq_(C_methyl_). The C12 methyl group exhibits orientational disorder of the hydrogen atoms. Two sets of H atoms were refined with occupancies of 0.56 (3) and 0.44 (3).

### Crystal data

**Table:**

C_13_H_15_NO_3_	*F*(000) = 992
*M**_r_* = 233.26	*D*_x_ = 1.221 Mg m^−^^3^
Monoclinic, *C*2/*c*	Mo *K*α radiation, λ = 0.71073 Å
Hall symbol: -C 2yc	Cell parameters from 17006 reflections
*a* = 10.8789 (3) Å	θ = 1.9–29.4°
*b* = 11.9095 (2) Å	µ = 0.09 mm^−^^1^
*c* = 20.1004 (5) Å	*T* = 295 K
β = 103.014 (2)°	Block, colourless
*V* = 2537.36 (10) Å^3^	0.56 × 0.48 × 0.35 mm
*Z* = 8	

### Data collection

**Table:**

Oxford Diffraction Xcalibur Ruby Gemini diffractometer	2386 independent reflections
graphite	2029 reflections with *I* > 2σ(*I*)
Detector resolution: 10.434 pixels mm^-1^	*R*_int_ = 0.023
ω scans	θ_max_ = 25.6°, θ_min_ = 2.1°
Absorption correction: analytical (*CrysAlis PRO*; Oxford Diffraction, 2009)	*h* = −13→13
*T*_min_ = 0.933, *T*_max_ = 0.965	*k* = −14→14
26868 measured reflections	*l* = −24→24

### Refinement

**Table:**

Refinement on *F*^2^	Secondary atom site location: difference Fourier map
Least-squares matrix: full	Hydrogen site location: inferred from neighbouring sites
*R*[*F*^2^ > 2σ(*F*^2^)] = 0.037	H-atom parameters constrained
*wR*(*F*^2^) = 0.102	*w* = 1/[σ^2^(*F*_o_^2^) + (0.0469*P*)^2^ + 1.3266*P*] where *P* = (*F*_o_^2^ + 2*F*_c_^2^)/3
*S* = 1.04	(Δ/σ)_max_ = 0.001
2386 reflections	Δρ_max_ = 0.17 e Å^−^^3^
159 parameters	Δρ_min_ = −0.12 e Å^−^^3^
0 restraints	Extinction correction: *SHELXL97* (Sheldrick, 2008), Fc^*^=kFc[1+0.001xFc^2^λ^3^/sin(2θ)]^-1/4^
Primary atom site location: structure-invariant direct methods	Extinction coefficient: 0.0142 (9)

### Special details

**Table:**

Geometry. All e.s.d.'s (except the e.s.d. in the dihedral angle between two l.s. planes) are estimated using the full covariance matrix. The cell e.s.d.'s are taken into account individually in the estimation of e.s.d.'s in distances, angles and torsion angles; correlations between e.s.d.'s in cell parameters are only used when they are defined by crystal symmetry. An approximate (isotropic) treatment of cell e.s.d.'s is used for estimating e.s.d.'s involving l.s. planes.
Refinement. Refinement of *F*^2^ against ALL reflections. The weighted *R*-factor *wR* and goodness of fit *S* are based on *F*^2^, conventional *R*-factors *R* are based on *F*, with *F* set to zero for negative *F*^2^. The threshold expression of *F*^2^ > σ(*F*^2^) is used only for calculating *R*-factors(gt) *etc*. and is not relevant to the choice of reflections for refinement. *R*-factors based on *F*^2^ are statistically about twice as large as those based on *F*, and *R*- factors based on ALL data will be even larger.

### Fractional atomic coordinates and isotropic or equivalent isotropic displacement parameters (Å^2^)

**Table:**

	*x*	*y*	*z*	*U*_iso_*/*U*_eq_	Occ. (<1)
C1	0.35967 (13)	0.14861 (11)	0.17051 (7)	0.0419 (3)	
C2	0.28501 (17)	0.17469 (12)	0.22158 (8)	0.0576 (4)	
H2	0.2547	0.2478	0.2206	0.069*	
C3	0.25517 (19)	0.10848 (13)	0.26854 (8)	0.0634 (5)	
H3	0.2108	0.1445	0.2968	0.076*	
C4	0.27916 (15)	−0.01212 (12)	0.28450 (7)	0.0486 (4)	
C5	0.42558 (11)	0.23129 (11)	0.07158 (6)	0.0379 (3)	
C6	0.51419 (13)	0.31543 (12)	0.06931 (7)	0.0456 (3)	
C7	0.56646 (14)	0.31987 (13)	0.01267 (8)	0.0542 (4)	
H7	0.6248	0.376	0.0104	0.065*	
C8	0.53555 (14)	0.24425 (13)	−0.04058 (8)	0.0523 (4)	
C9	0.44899 (14)	0.16118 (12)	−0.03596 (7)	0.0490 (4)	
H9	0.4288	0.1084	−0.0708	0.059*	
C10	0.39115 (12)	0.15365 (11)	0.01878 (7)	0.0402 (3)	
C11	0.55135 (18)	0.39999 (15)	0.12605 (9)	0.0693 (5)	
H11A	0.6218	0.4432	0.119	0.104*	
H11B	0.5744	0.3615	0.169	0.104*	
H11C	0.4815	0.4491	0.1263	0.104*	
C12	0.5918 (2)	0.25320 (19)	−0.10259 (10)	0.0801 (6)	
H12A	0.5606	0.1931	−0.1336	0.12*	0.56 (3)
H12B	0.6821	0.2483	−0.0887	0.12*	0.56 (3)
H12C	0.5686	0.3239	−0.1248	0.12*	0.56 (3)
H12D	0.6469	0.3171	−0.0978	0.12*	0.44 (3)
H12E	0.5255	0.2619	−0.1428	0.12*	0.44 (3)
H12F	0.6389	0.1863	−0.1066	0.12*	0.44 (3)
C13	0.29160 (14)	0.06565 (12)	0.01791 (8)	0.0519 (4)	
H13A	0.2615	0.0387	−0.0279	0.078*	
H13B	0.2227	0.0977	0.034	0.078*	
H13C	0.327	0.0044	0.047	0.078*	
N1	0.36385 (11)	0.23264 (9)	0.12731 (6)	0.0424 (3)	
H1N	0.3257	0.2937	0.1335	0.051*	
O1	0.41359 (10)	0.05741 (8)	0.16796 (5)	0.0508 (3)	
O2	0.34725 (10)	−0.07126 (8)	0.25195 (5)	0.0521 (3)	
H2A	0.3763	−0.0267	0.2227	0.062*	
O3	0.23146 (13)	−0.05442 (9)	0.32755 (6)	0.0693 (4)	

### Atomic displacement parameters (Å^2^)

**Table:**

	*U*^11^	*U*^22^	*U*^33^	*U*^12^	*U*^13^	*U*^23^
C1	0.0520 (8)	0.0339 (7)	0.0424 (7)	0.0010 (6)	0.0165 (6)	0.0039 (5)
C2	0.0900 (12)	0.0343 (7)	0.0601 (9)	0.0118 (7)	0.0415 (9)	0.0074 (6)
C3	0.1035 (13)	0.0422 (8)	0.0594 (10)	0.0066 (8)	0.0497 (10)	0.0029 (7)
C4	0.0692 (9)	0.0397 (7)	0.0379 (7)	−0.0098 (7)	0.0143 (7)	0.0026 (6)
C5	0.0387 (7)	0.0361 (7)	0.0401 (7)	−0.0004 (5)	0.0114 (5)	0.0075 (5)
C6	0.0452 (8)	0.0414 (7)	0.0498 (8)	−0.0081 (6)	0.0098 (6)	0.0039 (6)
C7	0.0474 (8)	0.0531 (9)	0.0658 (10)	−0.0150 (7)	0.0209 (7)	0.0091 (7)
C8	0.0515 (8)	0.0575 (9)	0.0531 (9)	−0.0009 (7)	0.0225 (7)	0.0095 (7)
C9	0.0556 (9)	0.0491 (8)	0.0440 (8)	−0.0036 (7)	0.0149 (6)	−0.0009 (6)
C10	0.0402 (7)	0.0364 (7)	0.0441 (7)	−0.0030 (5)	0.0099 (6)	0.0052 (5)
C11	0.0781 (12)	0.0590 (10)	0.0706 (11)	−0.0267 (9)	0.0163 (9)	−0.0111 (8)
C12	0.0877 (13)	0.0929 (15)	0.0740 (12)	−0.0034 (11)	0.0480 (11)	0.0131 (10)
C13	0.0547 (9)	0.0453 (8)	0.0546 (9)	−0.0148 (7)	0.0101 (7)	0.0023 (6)
N1	0.0534 (7)	0.0323 (6)	0.0459 (6)	0.0032 (5)	0.0204 (5)	0.0065 (5)
O1	0.0653 (6)	0.0409 (5)	0.0518 (6)	0.0141 (5)	0.0252 (5)	0.0110 (4)
O2	0.0692 (7)	0.0352 (5)	0.0546 (6)	−0.0021 (5)	0.0197 (5)	0.0072 (4)
O3	0.1074 (10)	0.0511 (7)	0.0589 (7)	−0.0122 (6)	0.0390 (7)	0.0112 (5)

### Geometric parameters (Å, °)

**Table:**

C1—O1	1.2409 (16)	C9—C10	1.3880 (19)
C1—N1	1.3325 (16)	C9—H9	0.93
C1—C2	1.4784 (19)	C10—C13	1.5044 (18)
C2—C3	1.325 (2)	C11—H11A	0.96
C2—H2	0.93	C11—H11B	0.96
C3—C4	1.482 (2)	C11—H11C	0.96
C3—H3	0.93	C12—H12A	0.96
C4—O3	1.2140 (17)	C12—H12B	0.96
C4—O2	1.3004 (18)	C12—H12C	0.96
C5—C10	1.3937 (19)	C12—H12D	0.96
C5—C6	1.3983 (18)	C12—H12E	0.96
C5—N1	1.4300 (16)	C12—H12F	0.96
C6—C7	1.383 (2)	C13—H13A	0.96
C6—C11	1.507 (2)	C13—H13B	0.96
C7—C8	1.381 (2)	C13—H13C	0.96
C7—H7	0.93	N1—H1N	0.86
C8—C9	1.383 (2)	O2—H2A	0.9
C8—C12	1.511 (2)		
			
O1—C1—N1	123.00 (12)	C9—C10—C13	119.30 (13)
O1—C1—C2	123.46 (12)	C5—C10—C13	122.71 (12)
N1—C1—C2	113.55 (12)	C6—C11—H11A	109.5
C3—C2—C1	129.10 (14)	C6—C11—H11B	109.5
C3—C2—H2	115.5	H11A—C11—H11B	109.5
C1—C2—H2	115.5	C6—C11—H11C	109.5
C2—C3—C4	132.18 (14)	H11A—C11—H11C	109.5
C2—C3—H3	113.9	H11B—C11—H11C	109.5
C4—C3—H3	113.9	C8—C12—H12A	109.5
O3—C4—O2	121.14 (14)	C8—C12—H12B	109.5
O3—C4—C3	118.28 (14)	C8—C12—H12C	109.5
O2—C4—C3	120.56 (12)	C8—C12—H12D	109.5
C10—C5—C6	121.31 (12)	C8—C12—H12E	109.5
C10—C5—N1	120.72 (11)	H12D—C12—H12E	109.5
C6—C5—N1	117.79 (12)	C8—C12—H12F	109.5
C7—C6—C5	117.93 (13)	H12D—C12—H12F	109.5
C7—C6—C11	120.49 (13)	H12E—C12—H12F	109.5
C5—C6—C11	121.58 (13)	C10—C13—H13A	109.5
C8—C7—C6	122.64 (13)	C10—C13—H13B	109.5
C8—C7—H7	118.7	H13A—C13—H13B	109.5
C6—C7—H7	118.7	C10—C13—H13C	109.5
C7—C8—C9	117.71 (13)	H13A—C13—H13C	109.5
C7—C8—C12	121.33 (15)	H13B—C13—H13C	109.5
C9—C8—C12	120.95 (15)	C1—N1—C5	126.37 (11)
C8—C9—C10	122.43 (14)	C1—N1—H1N	116.8
C8—C9—H9	118.8	C5—N1—H1N	116.8
C10—C9—H9	118.8	C4—O2—H2A	109.5
C9—C10—C5	117.95 (12)		
			
O1—C1—C2—C3	−4.9 (3)	C7—C8—C9—C10	1.6 (2)
N1—C1—C2—C3	175.19 (19)	C12—C8—C9—C10	−176.99 (15)
C1—C2—C3—C4	−3.2 (4)	C8—C9—C10—C5	−2.0 (2)
C2—C3—C4—O3	−174.0 (2)	C8—C9—C10—C13	175.94 (14)
C2—C3—C4—O2	4.5 (3)	C6—C5—C10—C9	0.9 (2)
C10—C5—C6—C7	0.4 (2)	N1—C5—C10—C9	176.00 (12)
N1—C5—C6—C7	−174.81 (12)	C6—C5—C10—C13	−176.92 (13)
C10—C5—C6—C11	179.69 (14)	N1—C5—C10—C13	−1.86 (19)
N1—C5—C6—C11	4.5 (2)	O1—C1—N1—C5	1.9 (2)
C5—C6—C7—C8	−0.8 (2)	C2—C1—N1—C5	−178.23 (13)
C11—C6—C7—C8	179.93 (16)	C10—C5—N1—C1	59.84 (18)
C6—C7—C8—C9	−0.2 (2)	C6—C5—N1—C1	−124.94 (15)
C6—C7—C8—C12	178.41 (16)		

### Hydrogen-bond geometry (Å, °)

**Table:**

*D*—H···*A*	*D*—H	H···*A*	*D*···*A*	*D*—H···*A*
O2—H2A···O1	0.90	1.61	2.5037 (13)	174
N1—H1N···O3^i^	0.86	2.12	2.9587 (15)	165

Symmetry codes: (i) −*x*+1/2, *y*+1/2, −*z*+1/2.
